# Characterization of manganized soluble dietary fiber complexes from tigernut meal and study of the suppressive activity of digestive enzymes *in vitro*

**DOI:** 10.3389/fnut.2023.1157015

**Published:** 2023-05-05

**Authors:** Yifei Wang, Weihao Wang, Yunjiao Wu, Junlan JiLiu, Xin Hu, Mingzhi Wei, LongKui Cao

**Affiliations:** ^1^College of Food Science, Heilongjiang Bayi Agricultural University, Daqing, China; ^2^National Coarse Cereals Engineering Research Center, Heilongjiang Bayi Agricultural University, Daqing, China

**Keywords:** soluble dietary fiber, manganese, structural characterization, α-amylase, α-glucosidase

## Abstract

In this study, manganized soluble dietary fiber (SDF–Mn(II)) was prepared from tigernut meal using a microwave solid-phase synthesis method with SDF. Microscopic morphological and structural analyses of SDF–Mn(II) were carried out using scanning electron microscopy, Fourier infrared spectroscopy, UV full-band scanning, X-ray diffraction, a thermal analyzer, gel permeation chromatography, and nuclear magnetic resonance, and its *in vitro* hypoglycemic activity was initially investigated. The results of these analyses revealed that the reaction of Mn(II) with SDF mainly involved hydroxyl and carbonyl groups, with the Nuclear magnetic resonance (NMR) analysis showing that specific covalent binding was produced and substitution was mainly carried out at the C_6_ position. Moreover, compared with SDF, the SDF–Mn(II) complex exhibited a porous structure, red-shifted, and color-enhancing effects on the UV characteristic peaks, significantly increased crystallinity and decreased molecular weight, and improved thermal stability; in addition, SDF–Mn(II) afforded significantly enhanced inhibition of α-amylase and α-glucosidase and possesses good *in vitro* digestive enzyme inhibition activity.

## 1. Introduction

Tigernuts (*Cyperus esculentus* L.) are widely distributed throughout the world, mainly as a snack in tropical and Mediterranean regions. They are often used in the food industry for the production of flavored beverages (1, 2). They have the ability to tolerate drought and sandy and acidic environments and are now widely grown in the northern regions of China (3), where they are cultivated with great ability and high yield. Because of its high oil content and ease of cultivation, it has potential value for the development of edible oil resources in China. Tigernut meal, a by-product of the processing of tigernuts, is rich in dietary fiber and a good source of high-quality dietary fiber.

The intake of dietary fiber is inversely proportional to the level of blood glucose values, and many studies have shown that a moderate intake of dietary fiber can prevent the development of diabetes and alleviate the manifestations of the disease, to some extent, in these patients (4). Dietary fiber lowers blood glucose mainly by improving insulin resistance, regulating disorders of glucolipid metabolism, improving oxidative stress and the inflammatory response, and regulating the intestinal flora; furthermore, it stabilizes postprandial blood glucose by inhibiting the activity of digestive enzymes and delaying glucose absorption in the intestine (5).

Manganese is an essential trace element that is mainly taken up through food and water, digested and absorbed through the gastrointestinal tract, and transported to mitochondria-rich organs (especially the liver, pancreas, and pituitary gland), where it exerts its biological effects (6). Manganese is involved in the synthesis and activation of various enzymes in the body, aids in glucose and lipid metabolism, regulates endocrine disorders, and improves immune function (7). Manganese supplementation also increases insulin secretion, improves glucose tolerance under conditions of dietary stress, and prevents type II diabetes and its complications (8). A moderate intake of organic trace elements can improve animal productivity and immunity, with the advantage of being able to reduce antagonistic effects among trace elements (9). Previous studies have shown that heavy metals can denature enzymes, resulting in a decrease in enzymatic activity (10).

α-amylase and α-glucosidase are important enzymes in the catabolism of starch, glycogen, and disaccharides in the gastrointestinal tract. Because of the reduced rate of intestinal carbohydrate metabolism, inhibition of the activity of these enzymes is commonly used to control blood glucose levels (11). α-Glucosidase plays an important role in the regulation of postprandial blood glucose levels in humans (12), and its inhibitors block postprandial hyperglycemia and are commonly used to prevent or treat type II diabetes (13). α-amylase acts as a catalyst in reactions involving α-1,4-glycosidic bonds, to hydrolyze branched-chain starch, straight-chain starch glycogen, and many maltodextrins, thus acting as a catalyst in the reactions responsible for starch digestion (14).

Tigernut meal is a by-product of the processing of tigernuts, and there is no report on the inhibition of *in vitro* enzyme activity by chelation of SDF from tigernut meal with metal ions. In this study, the method of solid-state microwave synthesis is adopted, an SDF–Mn(II) complex was prepared by introducing Mn^2+^ (which is a factor that can increase insulin secretion) onto SDF (which has an anti-glycemic effect) using the latter as the raw material. The particle morphology, structural characterization, relative molecular mass, and thermal properties of SDF and SDF–Mn(II) were determined using scanning electron microscopy, Fourier transform infrared spectroscopy, ultraviolet spectroscopy, X-ray diffraction, NMR, gel permeation chromatography, and a thermal analyzer; moreover, their *in vitro* digestive-enzyme inhibitory activities were investigated to provide a new direction for controlling blood glucose levels and slowing down blood glucose elevation.

## 2. Materials and methods

### 2.1. Materials

Commercially available tigernut meal was used. α-amylase (enzymatic activity, 50 U/mg) and α-glucosidase (enzymatic activity, 40–80 U/mg) were purchased from Sigma, United States. Manganese chloride was from Tianjin Damao Chemical Reagent Factory. The remaining chemicals and reagents were of analytical grade.

### 2.2. Extraction of SDF from tigernut meal

The preparation of SDF from defatted tigernut meal was carried out according to the method of Shen et al. (15), with slight modification. The SDF was extracted from the supernatant by centrifugation, concentrated by rotary evaporation, subjected to alcoholic sedimentation in 95% ethanol for 12 h, and freeze dried after centrifugation for 8 h. The purified SDF was obtained by dialysis and deproteinization (16).

### 2.3. Synthesis of SDF–Mn(II) complexes

SDF–Mn(II) was synthesized according to the method of Xu Lockping (17). SDF and MnCl_2_ were weighed according to the mass ratio of 1:0.8, followed by the addition of 150% (relative to the mass of SDF) anhydrous ethanol; the solution was mixed well and placed in a microwave solid-phase synthesis extractor (Xianghu Technology Development Co., Ltd., Beijing, China), with the microwave time set to 3 min and its power set to 210 W for the coordination reaction. The precipitate was dried in a hot-air drying oven (55°C) to a constant weight, to obtain SDF–Mn(II).

### 2.4. Determination of manganese content and fit rate in SDF–Mn(II)

The obtained samples were dissolved and the content of manganese (II) was determined using a spectrophotometric method (540 nm) (18). The equation of the manganese standard curve was as follows: *y* = 0.0343*x* + 0.002, with a linear correlation coefficient of *R*^2^ = 0.9998, where y is the manganese content and x is the absorbance in μg/g. The manganese content of the SDF–Mn(II) prepared in this experiment was 71.89 μg/g, with a fit ratio of 41.60%.

### 2.5. Scanning electron microscopy (SEM) analysis

The microstructure of SDF and SDF–Mn(II) was observed using SEM (Type-SU1510 Hitachi microscope; HITACHI Inc., Japan) (19). The samples were dried and processed, and a specific amount was collected and bonded using conductive tape; the samples were then gold-plated and observed.

### 2.6. Fourier transform infrared (FT-IR) spectroscopy

FT-IR (Tensor 27 instrument; Bruker Daltonics Inc., Bremen, Germany) was used for the determination the sample (20). The sample was mixed with potassium bromide powder in the ratio of 1:100 and fully ground in a mortar, to homogenize the mixture, which was then poured into a compression device and finally scanned on the machine (4,000–400 cm^−1^).

### 2.7. UV spectroscopy

This experiment was performed using an ultraviolet generalizable spectrophotometer (T6 series; Yuan Analysis Instrument Co., Ltd., Shanghai, China). The sample solution was prepared at a mass concentration of 2 mg/ml, and distilled water was used as a blank control. The sample to be measured was aspirated using a syringe, filtered through a pinhole filter, and scanned in the wavelength range of 190–400 nm with a scan interval of 1 nm (21).

### 2.8. X-ray diffraction (XRD) pattern analysis

Measurements were performed using an X-ray diffractometer (Type D/MAX2000V, Neo-Confucianism Manufacturing Company, Japan). The dried and delicate samples were uniformly dispersed in the plate frame and compacted, so that the sample surface was smooth and flat, and the sample frame was fixed and tested. The diffraction test conditions were as follows: tube current, 40 mA; tube voltage, 40 kV; Cu target wavelength, 1.5406 Å; Co target wavelength, 1.79026 Å; scan rate, 7°/min; and measurement range, 2θ from 5° to 70° (22).

### 2.9. Molecular weight determination

A narrowly distributed polyethylene glycol (PEO) was used as the standard curve for the relative calibration method and as the standard sample group in the detection using a differential refractive index detector (RID-20, Shimadzu, Japan). The precipitate was washed twice with anhydrous ethanol, air dried, dissolved by adding a solution of 0.1 mol/l NaNO_3_ and 0.06% NaN_3_, reacted at 121°C for 20 min, and centrifuged at 5000 r/min for 10 min; subsequently, 20 μl of the sample was collected. The detection conditions were as follows: flow rate, 0.6 ml/min, and column temperature, 35°C.

### 2.10. Nuclear magnetic resonance (NMR) measurements

The samples were dissolved in D_2_O and shaken well to achieve complete dissolution, followed by 1D-NMR (1H-NMR, 13C-NMR) measurements using a 600 MHz NMR instrument (Bruker AVANCE III, Brooke, Inc., Germany) (23).

### 2.11. Thermal stability analysis

These measurements were performed using a thermogravimetric analyzer (TGA 550; TA Instruments, New Castle, DE, United States) (24). A 20.0 mg sample was placed in an alumina crucible and heated in the temperature range of 25°C–600°C at a rate of 10°C/min under a nitrogen atmosphere, to obtain TGA and DSC curves.

### 2.12. *In vitro* enzymatic activity inhibition study

#### 2.12.1. Inhibition of α-glucosidase by SDF and SDF–Mn(II)

α-Glucosidase was diluted with 0.1 mol/l (pH = 6.8) phosphate-buffered solution to 1 U/ml. For the assay, 50 μl of the sample solution and 50 μl of the pNPG solution were simultaneously added to a 96-well plate. Incubate at 37°C for 10 min, and then 100 μl of the α-glucosidase solution was added and incubated for 45 min at 37°C. The reaction was terminated by adding 50 μl of Na_2_CO_3_ solution at a concentration of 0.2 mol/l. The absorbance at 405 nm was measured using an enzyme standardizer for the calculation of the enzyme inhibition rate (25).

#### 2.12.2. Inhibition of α-amylase by SDF and SDF–Mn(II)

A buffer solution was used to prepare porcine α-amylase at a concentration of 2 U/ml. The sample solution at different concentration gradients was mixed with 40 μl of α-amylase and incubated at 37°C for 30 min. A 40 μl of soluble starch was then added and incubated for 10 min, followed by the addition of 160 μl of DNS and boiling for 5 min, for color development. The absorbance of the inhibited group was measured at 540 nm; control, background, and blank groups were also used in this experiment (26).

#### 2.12.3. Data statistics and analysis

Data were processed using the SPSS 22 software, and the statistical analysis of the data was performed using the Excel 2019 software, whereas plotting was performed using the Origin 96 software. Three groups of parallel experiments were set up for all experiments.

## 3. Results

### 3.1. SEM analysis

**Figure 1:**
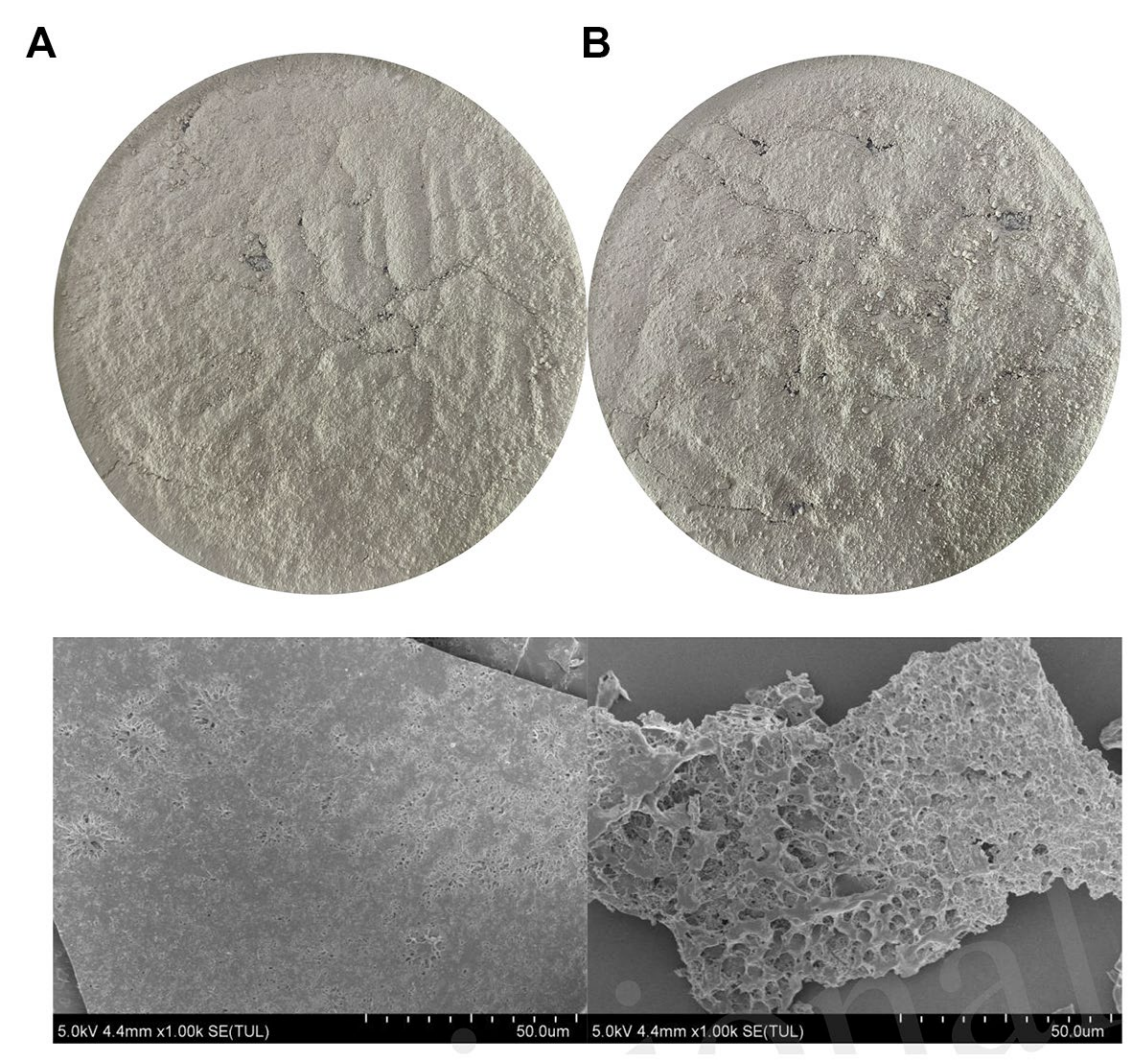
The SEM diagram of SDF **(A)**, SDF-Mn(II) **(B)**.

The results of the SEM analysis of SDF and SDF–Mn(II) are reported in Figure 1. From the figure, we can clearly see that SDF is in the form of a sheet structure, with a dense structure and fewer holes. However, SDF-Mn (II) structure presents a cellular structure with obvious fragmentation trend, which increases the relative surface area and may lead to changes in its physical and chemical properties. After microwave treatment, the internal structure, morphology, and polymerization mode were altered, and the wrapped groups were exposed, which laid the structural foundation for the full completion of the subsequent chelation reaction.

### 3.2. FT-IR analysis

**Figure 2:**
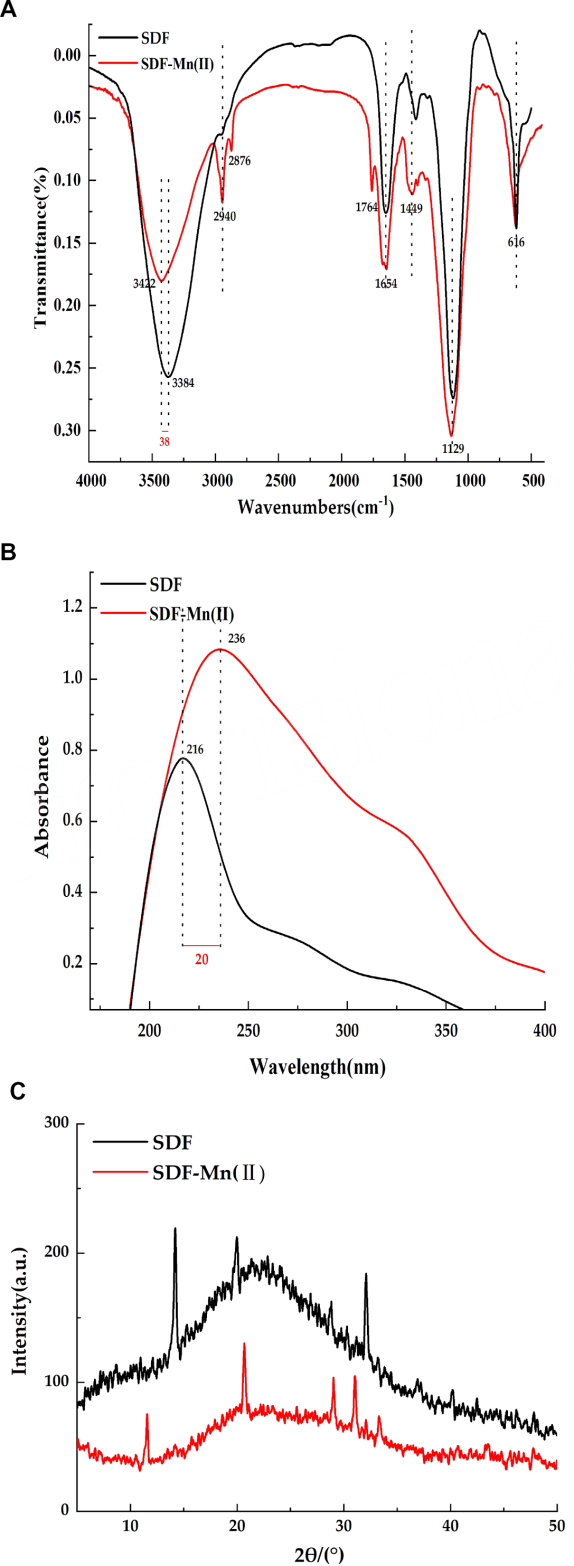
FT-IR spectrum diagram **(A)**, UV spectra **(B)**, X-ray diffraction plot **(C)**.

The Fourier transform infrared spectra of SDF and SDF–Mn(II) are shown in Figure 2A. SDF showed a characteristic absorption peak of O-H at 3,384 cm^−1^ (27), while the O-H characteristic absorption peak in the absorption spectrum of SDF-Mn(II) was red-shifted to 3,422 cm^−1^. The intensity of the SDF-Mn(II) absorption peak becomes weaker, which may be due to the chelation reaction consuming part of the O-H in SDF. The absorption peak detected at 2,940 cm^−1^ may be attributed to the C-H stretching vibration of the -CH_2_ group (28). In turn, the peak near 1,764 cm^−1^ in the SDF–Mn(II) spectrum may be attributed to the stretching vibration of the carbonyl group, which is not present in SDF (29); thus, the carbonyl group may be involved in the chelation reaction. SDF has a strong absorption peak near 1,654 cm^−1^ for the carboxyl group (30). The peak here in SDF-Mn(II) was not significantly shifted, but the peak strength weakened，indicating that the carboxyl group may be involved in the chelation reaction. The presence of an absorption peak near 1,449 cm^−1^ indicated the existence of a pyranoside functional group (31). The appearance of the absorption peak at 1,129 cm^−1^ was mainly attributed to the coupling valence vibration of the C=O bond and the deformation vibration of the C–H bond (32). Most of the characteristic peaks of SDF did not significantly change between before and after the modification, indicating that the basic skeleton of SDF remained unchanged. Finally, the IR spectrograms showed that the hydroxyl and carbonyl groups were mainly involved in the chelation reaction.

### 3.3. UV spectroscopy

The UV spectra of SDF and SDF–Mn(II) are provided in Figure 2B. There was no obvious absorption peak between 260 and 280 nm, indicating a negligible amount of protein in the sample (33). It can be seen from the figure that SDF shows a strong absorption peak at 216 nm, while SDF-Mn(II) shows a strong absorption peak at 236 nm. SDF–Mn(II) is a complex comprising several components. Mn^2+^ is an oxidation state transition metal ion with a half-full d orbital (an electron acceptor), and SDF is an organic compound with a conjugated π-electron system (an electron donor). Therefore, SDF–Mn(II) belongs to the spectral ligand-to-metal charge transfer, a process equivalent to the reduction of metals. With the enhancement of the metal cation reduction ability, the wavelength shifts toward the long wave direction, producing a red-shift effect. In turn, with the enhancement of the cation oxidation ability, the color deepens, producing a color-enhancing effect. Because the main chromogenic group present in SDF is the carbonyl group and the co-color group is the hydroxyl group (34), as can be seen from the figure, the absorption of SDF-Mn(II) in the UV region is significantly higher than that of SDF, thus indicating that mainly the carbonyl and hydroxyl groups are involved in the coordination reaction, which results in a change in the UV absorption intensity. It may also be due to the conjugation of several chromogenic groups to produce a new conjugated absorption band.

### 3.4. XRD analysis

The XRD analysis of SDF and SDF–Mn(II) is reported in Figure 2C. From the figure, we can see that both SDF and SDF-Mn(II) show broad diffraction peaks, which are typical for polymers, and the broad peaks indicate low crystallinity in the structure, which may be due to the fact that the extracted SDF is a mixed polysaccharide (35). Which are typical of polymers; moreover, wide peaks indicate a lower crystallinity in the structure, which may be attributed to the fact that the extracted SDF is a mixed polysaccharide. Furthermore, the figure shows that the peak dispersion of SDF was lower than that of SDF–Mn(II) and the calculated crystallinity of SDF was 23.48%, whereas that of SDF–Mn(II) was 33.82%, which may be attributed to the disruption of macromolecular chains after treatment, resulting in the higher crystallinity of SDF–Mn(II) (36, 37). The XRD results were very different, further confirming the formation of SDF–Mn(II).

### 3.5. Molecular weight analysis

The relative molecular masses of SDF and SDF–Mn(II) are provided in Table 1, from which it can be seen that the Mw of SDF was 5,776, with a dispersion coefficient of 25.72, whereas the Mw of SDF–Mn(II) was 2,567, with a dispersion coefficient of 4.25. The data included in the table revealed that the Mw and dispersion coefficient of SDF were increased and the molecular weight distribution broadened, whereas those of SDF–Mn(II) Mw were significantly reduced, probably because the molecular chains were opened after microwave treatment, resulting in a decrease in the molecular weight of the modified SDF; in contrast, the dispersion coefficient was also significantly reduced, which suggests that the modified SDF system is more homogeneous and simpler in composition (38).

**Table 1:** Relative molecular mass of SDF, SDF-Mn(II).

Index	SDF	SDF–Mn(II)
Mn(Da)	(2.25 ± 0.56) × 10^2^	(6.05 ± 1.23) × 10^2^
Mw(Da)	(5.776 ± 1.21) × 10^3^	(2.567 ± 1.57) × 10^3^
Mw/Mn	25.72 ± 0.23	4.25 ± 0.44

Mn, Number Average Molecular Weight; Mw, Weight Average Molecular Weight.

### 3.6. NMR analysis

**Figure 3:**
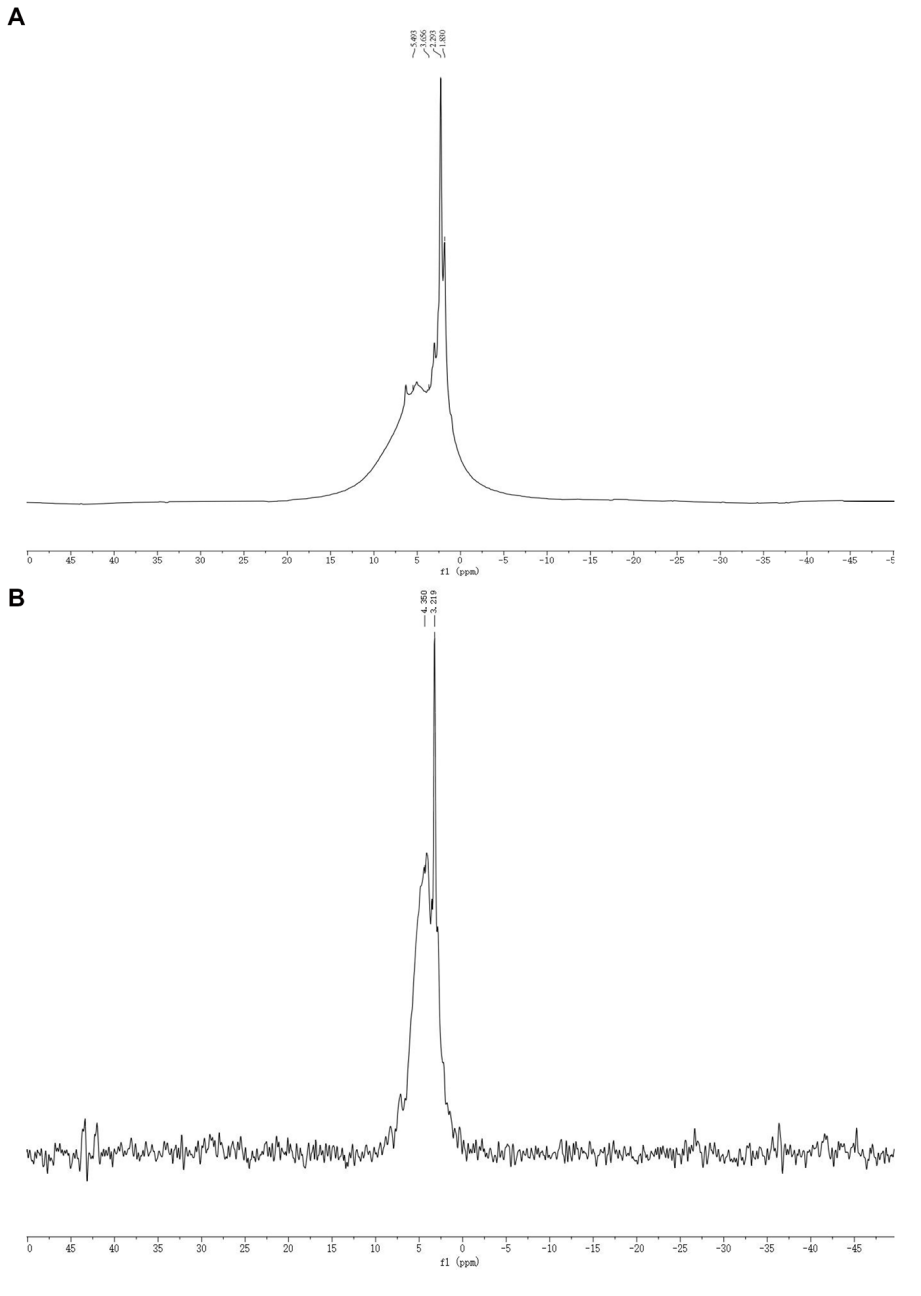
SDF **(A)**, SDF-Mn(II) **(B)** NMR ^1^H diagram.

The SDF and SDF–Mn(II) NMR ^1^H spectra are shown in Figure 3. The chemical shifts were affected by the sugar type, bond type, substitution, and modifications. It was previously shown that the chemical shifts are lower than 5.0 × 10^−6^ for β-configuration pyranosides and higher than 5.0 × 10^−6^ for α-configuration pyranosides, which can be used to distinguish the types of sugar rings (39). In the range of heteroheaded hydrogen proton signals, SDF exhibited three peaks at 1.830, 2.293, and 3.656 × 10^−6^, indicating that it belongs to the group of β-configuration pyranosides (40). In contrast, SDF–Mn(II) had two signal peaks at 3.219 and 4.350 × 10^−6^, indicating that the conformation of SDF did not significantly change after treatment and the reduced signal peak of SDF–Mn(II) may be attributed to the shortening of the ligand relaxation time that occurs upon binding of SDF to Mn^2+^; moreover, the large width of the signal range precluded the detection of the signal around paramagnetic Mn^2+^, thus forming a high-spin blind region with Mn^2+^ as the core (41).

**Figure 4:**
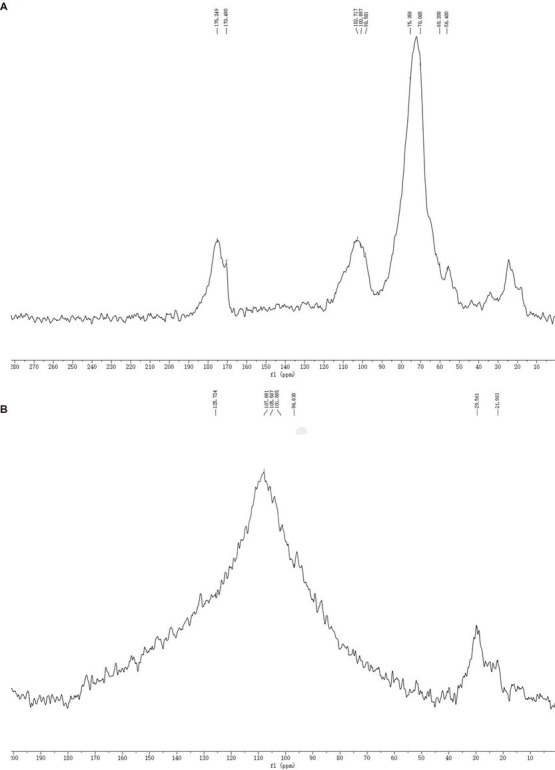
SDF **(A)**, SDF-Mn(II) **(B)** NMR ^13^C diagram.

The SDF and SDF–Mn(II) NMR ^13^C spectra are provided in Figure 4. The signal detected at 60.208 × 10^−6^ was attributed to the C_6_ glycosidic bond. The spectrum of SDF–Mn(II) became more complex because the carbon directly attached to the electron-absorbing group shifted to a lower field position, whereas the carbon indirectly attached to the electron-absorbing group shifted to a higher field position (42). Moreover, the signal peak of SDF–Mn(II) detected at 60.208 × 10^−6^ disappeared; this may be attributed to the highest -OH activity at the C_6_ position, which was replaced by the Mn^2+^ group. The C_1_ signal splits if the -OH on C_2_ is substituted, and this splitting correlates well with the degree of substitution on the C_2_ atom (43). In Figure 4B, At 90–100 × 10^−6^, the signal exhibited multiple splits, which may have been caused by the substitution of the hydroxyl group on C_2_ by the Mn^2+^ group (44), because the -OH activity at the C_2_ position was second only to that at C_6_.

### 3.7. Thermal characterization

**Figure 5:**
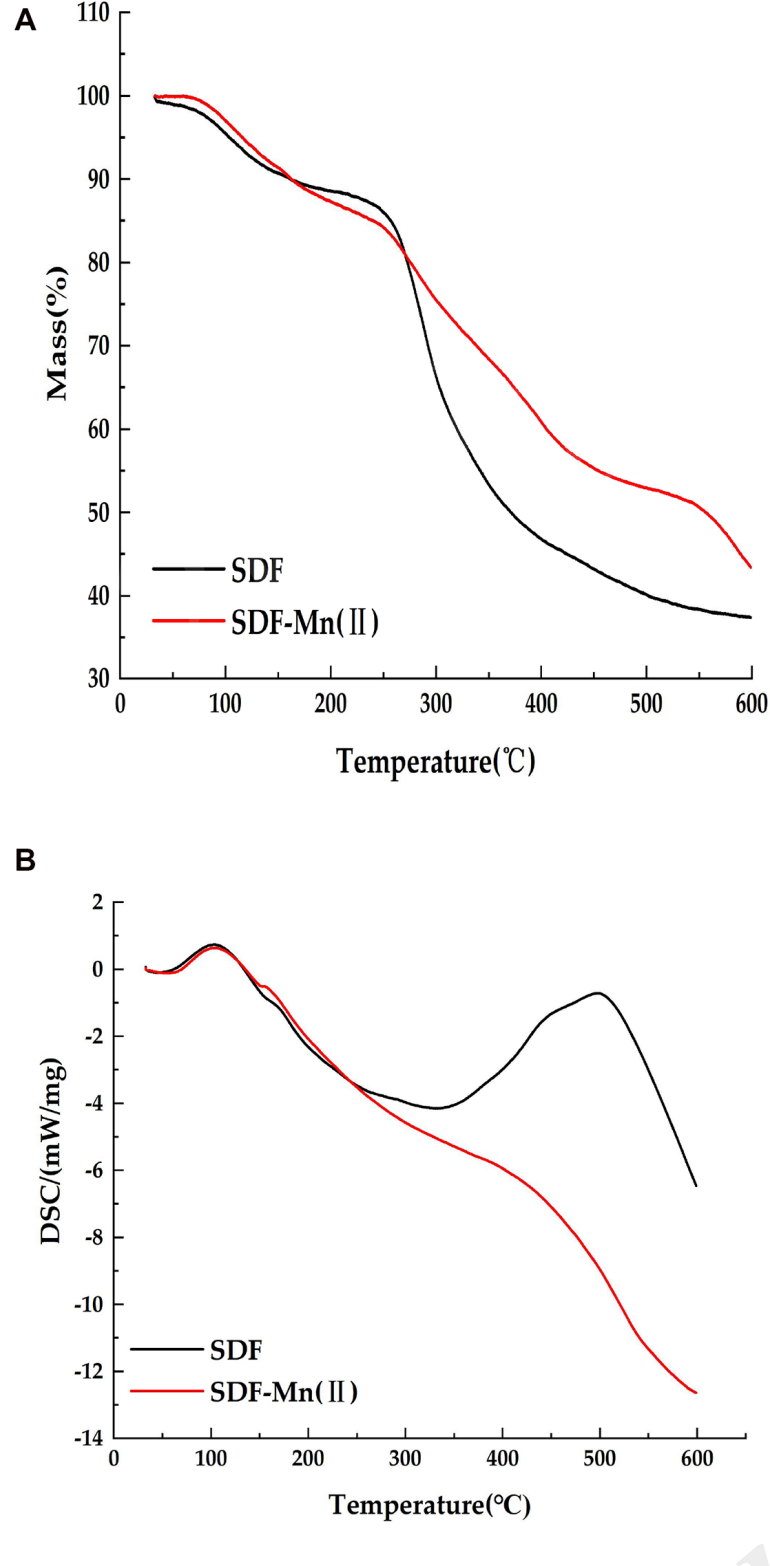
Thermal characteristics analysis of SDF, SDF–Mn(II). **(A)** The mass loss of SDF and SDF-Mn (II). **(B)** The changes in SDF and SDF-Mn (II) DSC.

Thermal stability plays an important role in food industry applications (45); therefore, the thermal properties of SDF before and after modification were characterized using TGA and DSC. Figure 5A shows that the decomposition temperature of SDF is 161°C, while the decomposition temperature of SDF-Mn(II) is 162°C, The two decomposition temperatures are similar, so the ease of dehydration is similar for both When the temperature increased from the decomposition temperature to 500°C, the weights of both samples started to significantly decrease because of the violent thermal degradation of the galacturonic acid chains in the samples, followed by decarboxylation of the acidic side groups in the rings and the carbon, which eventually produced different gaseous products, to form solid carbon (46, 47). Furthermore, the final residual mass of SDF (37.36%) was lower than that of SDF–Mn(II) (43.35%), suggesting that the thermal stability of SDF–Mn(II) is stronger than that of SDF. The curves of DSC presented in Figure 5B demonstrated that SDF had two exothermic peaks, at 105°C and 500°C, whereas SDF–Mn(II) exhibited one exothermic peak at 102°C. The appearance of two exothermic peaks for SDF may be related to the inhomogeneity of the composition, with SDF–Mn(II) becoming one peak, which suggests that the composition of SDF–Mn(II) is more homogeneous, in agreement with the results of the relative molecular mass analysis.

### 3.8. Inhibitory effect of SDF and SDF–Mn(II) on enzymatic activity *in vitro*

**Figure 6:**
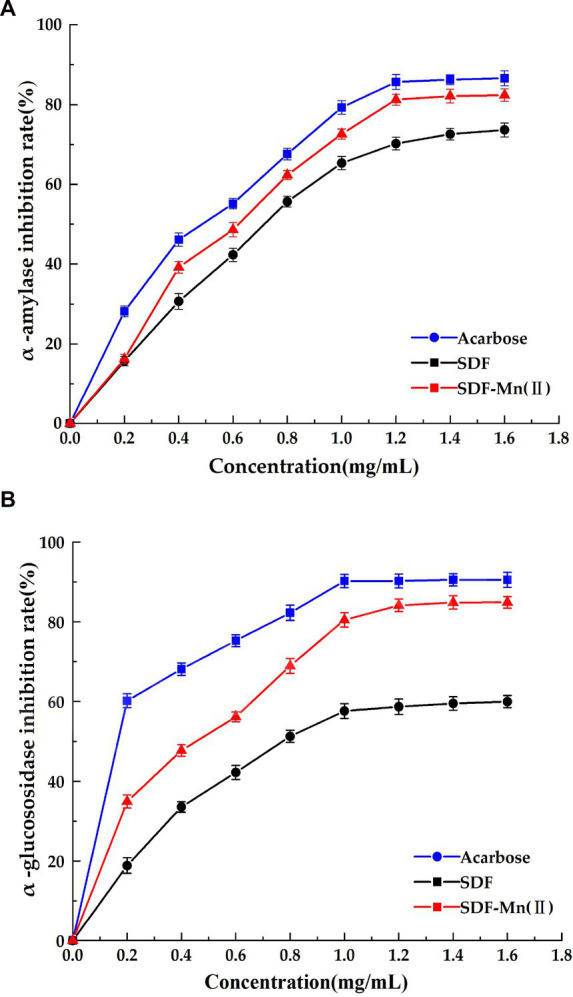
Effect of SDF and SDF-Mn(II) inhibition on α-amylase **(A)** and α-glucosidase **(B)**.

SDF has inhibitory activity against sugar hydrolases, including α-amylase and α-glucosidase; thus, it has the potential to replace commercial hypoglycemic drugs, such as acarbose and voglibose (48). The inhibition of α-amylase by SDF occurs via binding interactions between SDF and the active site of the enzymes as a result of hydrogen bonding and hydrophobic forces (44). The structure of SDF determines, to a large extent, its binding affinity to the enzymes. Figure 6 shows the inhibitory activities of SDF and SDF–Mn(II) toward α-amylase and α-glucosidase. Figure 6A shows the rate of inhibition of α-amylase, which gradually increases with increasing sample concentration (0–1.8 mg/ml); moreover, the inhibition rate of SDF–Mn(II) was stronger than that of SDF. When the sample concentration was greater than 1.2 mg/ml, the inhibitory effect no longer linearly increased, with the IC_50_ values of SDF and SDF–Mn(II) being 0.87 and 0.729 mg/ml, respectively. Figure 6B shows the rate of inhibition of α-glucosidase, which was similar to the results reported for α-amylase, with the SDF and SDF–Mn(II) IC_50_ values being 1.025 and 0.583 mg/ml, respectively. The figure demonstrated that there was a significant increase in the inhibitory rate of SDF–Mn(II) for both enzymes (49), which may be attributed to the microwave treatment, as it reduced the molecular weight of SDF–Mn(II) and facilitated binding to the active site of the enzyme, leading to an enhanced inhibition (50, 51); alternatively, the manganese element was introduced to denature the enzyme, leading to a decrease in enzymatic activity. The higher inhibitory activity of SDF–Mn(II) toward α-amylase and α-glucosidase may delay the absorption of dietary carbohydrates, which may contribute to the control of postprandial blood glucose levels.

## 4. Conclusion

This study reported the structural characterization and enzyme activity inhibition analysis of SDF and SDF–Mn(II). The experiments showed that SDF–Mn(II) exhibited a porous surface structure with a more obvious fragmentation trend, without obvious changes in the basic skeleton, as well as red-shifting and color-enhancing effects in the UV characteristic peaks, increased crystallinity, and decreased relative molecular mass. NMR revealed that SDF-Mn(II) mainly underwent a substitution reaction on C_6_. SDF-Mn(II) has better structural and thermal properties and has better inhibition of *in vitro* digestive enzymes, providing a good theoretical basis for further studies of SDF-Mn(II).

## Data availability statement

The original contributions presented in the study are included in the article/supplementary material, further inquiries can be directed to the corresponding author.

## Author contributions

WW and YuW: data curation. YiW, WW, YuW, and JJ: formal analysis. LC: funding acquisition. YiW and WW: investigation, methodology, and writing—original draft. YiW and JJ: validation. YiW, XH, MW, and LC: writing—review and editing. All authors contributed to the article and approved the submitted version.

## Funding

This work was supported by the Technology Major Program of Heilongjiang Province of Department of Science and Technology (2021ZX12B06), the National Key R&D Program of China (2021YFD2100902).

## Conflict of interest

The authors declare that the research was conducted in the absence of any commercial or financial relationships that could be construed as a potential conflict of interest.

## Publisher’s note

All claims expressed in this article are solely those of the authors and do not necessarily represent those of their affiliated organizations, or those of the publisher, the editors and the reviewers. Any product that may be evaluated in this article, or claim that may be made by its manufacturer, is not guaranteed or endorsed by the publisher.
